# Impact of insurance payer type (medicare vs. private) on the patient reported outcomes after shoulder arthroplasty

**DOI:** 10.1016/j.jseint.2024.08.199

**Published:** 2024-08-31

**Authors:** Paul V. Romeo, Aidan G. Papalia, Andrew J. Cecora, Bradley A. Lezak, Matthew G. Alben, Dashaun A. Ragland, Young W. Kwon, Mandeep S. Virk

**Affiliations:** a Division of Shoulder and Elbow Surgery, Department of Orthopedic Surgery, NYU Grossman School of Medicine, NYU Langone Orthopedic Hospital, NYU Langone Health, New York, NY, USA; b Department of Orthopedic Surgery, Rutgers Robert Wood Johnson School of Medicine, RWJ University Hospital, New Brunswick, NJ, USA; c Department of Orthopaedics and Sports Medicine, Jacobs School of Medicine and Biomedical Sciences, University at Buffalo, Buffalo, NY, USA

**Keywords:** Shoulder arthroplasty, Medicare, Private insurance, patient reported outcomes measurement information system upper extremity, Patient reported outcomes, ASES, SST

## Abstract

**Background:**

This study’s purpose is to determine if there is a difference in patient-reported outcome measures (PROMs) following shoulder arthroplasty (SA) based upon payer insurance type, with a secondary outcome of determining if any appreciable difference surpasses the minimal clinically important difference (MCID).

**Methods:**

Subjects undergoing anatomic and reverse total shoulder arthroplasty were prospectively enrolled between March 2019 and March 2021. Subjects completed patient reported outcomes measurement information system upper extremity (P-UE), the American Shoulder and Elbow Surgeons score (ASES), and the simple shoulder test (SST) preoperatively and at 2 weeks, 6 weeks, 3 months, 6 months, and 12 months, postoperatively. Descriptive statistics of baseline patient characteristics and preoperative PROMs (ASES, SST, and P-UE) were compared between insurance types.

**Results:**

143 patients were identified who met the inclusion criteria for this study. There were 98 patients within the Medicare cohort and 45 patients with private insurance. Patients in the Medicare cohort were older (mean age 70.5 vs. 61.3 years), with high proportion of smokers, diabetics, and reverse total shoulder arthroplasty compared to the private payor cohort. There were no significant differences between the two cohorts with respect to outcomes scores except for significantly better SST in the private insurance cohort (69.3 vs. 79.4, *P* = .02). No significant differences were noted for the achievement of MCID between cohorts [P-UE (*P* = 1.0), ASES (*P* = .25), and SST (0.52)] and pre-to-postoperative improvements for P-UE (*P* = .62), ASES (*P* = .4), or SST (0.66).

**Conclusion:**

Our study demonstrates that, at a tertiary-level academic institution in a metropolitan city, payor type does not have significant impact on achieving MCID or pre-to-postoperative improvements in PROMs after SA.

Shoulder arthroplasty (SA), inclusive of anatomic (aTSA) and reverse total shoulder arthroplasty (rTSA), is a common upper extremity (UE) arthroplasty procedure with about 53,000 cases performed each year. The number of SAs performed each year continues to increase with some projections expecting a 9-fold increase by 2030.^10^^,^^21^^,^^24^^,^^30^ The cause of the dramatic rise in the number of SA performed each year is multifactorial. First, the increased number of aging individuals in the United States leads to an increased prevalence of glenohumeral arthritis.^16^ Additionally, expanding indications of surgical innovations such as the rTSA has widened beyond rotator cuff tear arthropathy to include additional indications like primary glenohumeral arthritis with considerable glenoid wear, proximal humerus fractures, inflammatory arthropathy and failed primary aTSA.^18^^,^^25^^,^^36^^,^^39^

Given the increased prevalence of SA, it is imperative to understand all factors affecting patient care of individuals undergoing this surgery. This includes factors beyond the scope of clinical characteristics of patients, including factors such as the effect of payor type (insurance carrier; private vs. Medicare) on patient-reported outcomes measures (PROMs) and complications.^4^^,^^13^^,^^37^^,^^38^ In doing so, many policies, and consequently, clinical practice guidelines are driven by patient reported outcomes (PROMs) related to the specific intervention in question. PROMs allow clinicians and insurance providers alike the opportunity to further understand and assess the impact an orthopedic intervention has on a patient’s quality of life postoperatively.^2^^,^^12^^,^^20^ Currently, there is a limited literature examining the impact the type of insurance payor (ie, Medicare vs. private insurance) has on patient outcomes with regard to SA.^17^^,^^28^^,^^29^^,^^34^

The purpose of this study is to further evaluate and expand upon the literature regarding the implications different payor models have on PROMs by investigating the impact Medicare and private insurance have on patient reported outcomes measurement information system upper extremity (P-UE) version 2.0 CAT, ASES, and simple shoulder test (SST). The secondary purpose of this study is to determine if the score differences achieved the minimal clinical important difference (MCID) from pre-to-postoperatively. This value is associated with the minimal score difference that is associated with a clinical patient appreciable difference based on a patient’s own perception.^2^^,^^12^^,^^23^^,^^26^^,^^31^^,^^33^

## Methods

### Ethics

Internal institutional review board approval was granted for this study (Study ID s18-01216). All subjects provided informed consent prior to enrollment.

### Subject selection

Subjects undergoing SA (rTSA and aTSA) in the shoulder and elbow division at our institution were prospectively enrolled between March 2019 and March 2021. Subjects were consented for enrollment if they met all the following inclusion criteria: (1) 18 years of age or older, (2) preoperatively completed P-UE, ASES, and SST surveys, (3) demonstrated mental capacity, and (4) completed all surveys at a minimum of preoperatively, 6 months postoperatively and 12 months postoperatively. All surgeries were performed by one of the three shoulder fellowship- trained orthopedic surgeons at our academic institution. Patients were excluded from the study if they failed to meet the aforementioned criteria, had deceased before the 12-month follow-up visit, or declined to participate in the study. Demographics and socioeconomic characteristics were independently collected by two independently trained research assistants from electronic medical record review. Demographic data collected and evaluated include age, sex, marital status, postal code, social deprivation index, smoking status, body mass index, American Society of Anesthesiologists score , diagnosis, medical comorbidities, and race. Radiographs and computed tomographic images were reviewed by a shoulder and elbow fellowship-trained surgeon, and the glenoids were classified according to the Walch grading scale.^5^

### Patient-reported outcome measures

All subjects completed P-UE, ASES, and SST surveys. P-UE employs a T-score metric, normalized across a scale of 0-100, where the reference population mean is 50, with a standard deviation of 10.^11^^,^^12^^,^^15^ Higher scores on P-UE correlate with better UE function. ASES scores were obtained to evaluate both pain and function from patients when utilizing their shoulder.^32^ It is scored on a scale of 0-100, with zero denoting worse shoulder function and a score of 100 correlating with exceptional shoulder function.^32^ The SST questionnaire consists of twelve dichotomous questions (yes or no) with a score between zero and twelve. Worse shoulder function is associated with a score of zero, while excellent shoulder function is associated with a score of 12.^27^

### Survey and data collection

Subjects enrolled in our study were asked to complete five surveys, including P-UE, ASES, and SST, via email preoperatively, at 2 weeks, 6 weeks, 3 months, 6 months, and 12 months, postoperatively. Every subject completed a minimum of the preoperative, 6-month postoperative and 12-month postoperative surveys. Surveys were administered and monitored for completion via our institutionally hosted REDCap electronic data capture software (Vanderbilt University, Nashville, TN, USA). Phone surveys were available for patients unwilling or unable to complete email surveys. No significant difference between telephonic and email survey response scores has been demonstrated by previous investigations.^1^^,^^6^ Patients were stratified into Medicare vs. private insurance cohorts based upon primary payor.

### Statistical analysis

All statistical analysis was performed in Jupyter Lab using Python scripting (Project Jupyter, New York, NY, USA). Descriptive statistics of baseline patient characteristics and preoperative PROMs (ASES, SST, and P-UE) were compared between insurance types. Postoperative PROMS were compared by insurance type at each postoperative time point. Improvements in PROMs from baseline to 1-year postoperatively were determined for each patient. For each PROM, patient improvements were categorized as either “clinically significant” or “not clinically significant” based on whether or not MCID was achieved. This provided a threshold by which equivalence could be determined. MCIDs for ASES, SST, and P-UE were 20.9, 2.4, and 8.05, respectively, as established by previous literature.^12^^,^^35^ The number of patients achieving MCID for each PROM were compared between cohorts. For all statistical analyses, a *P* value <.05 was determined to be statistically significant.

## Results

### Demographics

**Figure 1:**
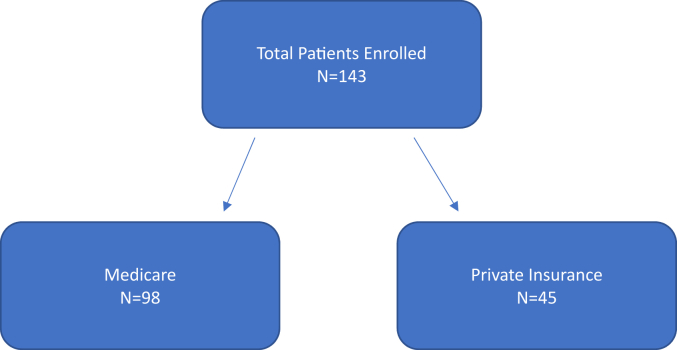
Patient cohorts.

A total of 178 patients were enrolled, and 143 patients (50.3% female; mean age of 67 ± 8 year old) met the inclusion criteria for this study. The excluded patients failed to meet the minimum follow-up at 6 and 12 months. 98 patients (56.1% female; mean age of 70 ± 7) were included in the Medicare cohort, and 45 patients (37.8% female; mean age of 61 ± 7) were included in the private insurance cohort (Fig. 1). Comprehensive patient demographics are presented in Table I. Patients in the Medicare cohort had higher proportion of smokers, and diabetics, and Charlson Comorbidity Index (CCI) was higher in the Medicare cohort compared to the private payor cohort (Table I).

**Table I:** Patient demographics.

	Overall, n = 143	Medicare, n = 98	Private, n = 45	*P* value
Age, mean (SD)	67.6 (8.2)	70.5 (7.0)	61.3 (7.2)	<.001
Sex, n (%)				.063
Female	72 (50.3)	55 (56.1)	17 (37.8)	
Male	71 (49.7)	43 (43.9)	28 (62.2)	
BMI, mean (SD)	29.7 (6.4)	29.2 (5.9)	31.0 (7.3)	.155
Race, n (%)				.977
Black, African American	7 (4.9)	5 (5.1)	2 (4.4)	
Hispanic	12 (8.4)	8 (8.2)	4 (8.9)	
White	124 (86.7)	85 (86.7)	39 (86.7)	
ASA, n (%)				.219
1	2 (1.4)	2 (2.0)		
2	105 (73.4)	68 (69.4)	37 (82.2)	
3	36 (25.2)	28 (28.6)	8 (17.8)	
Smoking status, n (%)				.01
Non-Smoker	68 (47.6)	39 (39.8)	29 (64.4)	
Smoker	75 (52.4)	59 (60.2)	16 (35.6)	
Charlson Comorbidity Index, mean (SD)	3.3 (1.74)	3.6 (1.72)	2.8 (1.66)	.01
DM, n (%)	36 (25.2)	30 (30.6)	6 (13.3)	.045
HLD, n (%)	74 (51.7)	55 (56.1)	19 (42.2)	.172
HTN, n (%)	85 (59.4)	56 (57.1)	29 (64.4)	.521
SDI percentile, n (%)				.117
<25th	24 (16.8)	20 (20.4)	4 (8.9)	
25-50th	47 (32.9)	35 (35.7)	12 (26.7)	
50-75th	70 (49.0)	42 (42.9)	28 (62.2)	
>75th	2 (1.4)	1 (1.0)	1 (2.2)	
rTSA vs. aTSA, n (%)				<.001
aTSA	27 (18.9)	10 (10.2)	17 (37.8)	
rTSA	116 (81.1)	88 (89.8)	28 (62.2)	

*BMI*, body mass index; *ASA*, American Society of Anesthesiologists score; *DM*, diabetes mellitus; *HLD*, hyperlipidemia; *HTN*, hypertension; *SDI*, social deprivation index; *rTSA*, reverse total shoulder arthroplasty; *aTSA*, anatomic total shoulder arthroplasty; *SD*, standard deviation.

Patients within the private payor cohort had a higher frequency of aTSA (*P* < .001), while those in the Medicare cohort displayed a higher frequency of rTSA (*P* < .001) (Table I). The Medicare cohort had a significantly higher proportion of patients with osteoarthritis and rotator cuff tears compared to patients in the private payor cohort, where there was a higher proportion of patients with primary osteoarthritis (Table II). There was no difference between the two cohorts with respect to glenoid wear pattern (Table II).

**Table II:** Procedure indications and complications.

	Overall, n = 143	Medicare, n = 98	Private, n = 45	*P* value
Etiology, n (%)				.03
Osteoarthritis with rotator cuff Tear	35 (24.4%)	31 (31.6%)	4 (8.8%)	
Primary osteoarthritis	68 (47.6%)	41 (41.8%)	27 (60.0%)	
Rotator cuff tear arthropathy	27 (18.9%)	18 (18.4%)	9 (20.0%)	
Revision shoulder arthroplasty	9 (6.3%)	4 (4.1%)	5 (11.1%)	
Proximal humerus fracture	2 (1.4%)	2 (2.0%)	0 (0%)	
Avascular necrosis	1 (0.7%)	1 (1.0%)	0 (0%)	
Instability arthropathy	1 (0.7%)	1 (1.0%)	0 (0%)	
Walch classification∗, %				.79
A1	4.9%	5.1%	4.4%	
A2	2.8%	3.1%	2.2%	
B1	35.0%	35.7%	33.3%	
B2	43.4%	45.9%	37.7%	
B3	2.8%	4.1%	0%	
C	0%	0%	0%	
D	0%	0%	0%	
3-month complication, n (%)	2 (1.4)	2 (2.0)	0 (0)	.345
3-month reoperation, n (%)	2 (1.4)	2 (2.0)	0 (0)	.345

∗ 11.2% of overall Walch classification unreported.

### Functional outcomes scores

Preoperative survey results showed no difference among Medicare and private payor cohorts for P-UE (*P* = .1), ASES (0.2), and SST (0.08). Furthermore, no difference in postoperative surveys was noted between the two cohorts at the 12-month follow-up for P-UE (*P* = .1) or ASES (*P* = .69). The private payor cohort had higher SST survey scores compared to the Medicare cohort (69.3 vs. 79.4, *P* = .02). No difference was noted between the two cohorts with respect to achievement of MCID for all PROMs [P-UE (*P* = 1.0), ASES (*P* = .25), and SST (0.52)].^12^^,^^35^ Each cohort displayed improvement from pre-to-postoperative period for P-UE (*P* < .001), ASES (*P* < .001), and SST (*P* < .001). No significant difference in pre-to-postoperative changes were noted between the two cohorts for either P-UE (*P* = .62), ASES (*P* = .41), or SST (0.66) (Tables III and IV).

**Table III:** Pre- and postoperative outcomes.

	Overall n = 143	Medicare n = 98	Private n = 45	*P* value
Preoperative score, mean (SD)				
P-UE	30.1 (6.3)	29.4 (6.1)	31.4 (6.5)	.099
ASES	40.3 (17.8)	38.9 (16.9)	43.3 (19.5)	.198
SST	28.9 (22.0)	26.6 (21.2)	33.9 (23.3)	.078
12-month postoperative score, mean (SD)				
P-UE	42.8 (8.4)	41.9 (7.8)	44.7 (9.6)	.098
ASES	81.4 (18.1)	81.0 (16.9)	82.4 (20.5)	.687
SST	72.5 (24.0)	69.3 (23.7)	79.4 (23.6)	.022
Pre-to-postoperative difference, mean (SD)				
P-UE	12.8 (8.7)	12.5 (8.2)	13.3 (9.7)	.625
ASES	41.4 (21.2)	42.4 (20.9)	39.1 (22.0)	.406
SST	43.1 (27.7)	42.4 (26.8)	44.7 (29.8)	.660
12-month MCID achieved, n (%)				
P-UE	101 (70.6)	69 (70.4)	32 (71.1)	1.000
ASES	123 (86.0)	87 (88.8)	36 (80.0)	.252
SST	127 (91.4)	88 (92.6)	39 (88.6)	.519

*P-UE*, patient reported outcomes measurement information system; Upper Extremity; *ASES*, American Shoulder and Elbow Surgeons score; *SST*, Simple Shoulder Test; *SD*, standard deviation; *MCID*, minimal clinically important difference.

**Table IV:** Pre-to-postoperative outcomes.

	Overall n = 143	Medicare n = 98	Private n = 45
Preoperative P-UE (SD)	30.1 (6.3)	29.4 (6.1)	31.4 (6.5)
Postoperative P-UE (SD)	42.8 (8.4)	41.9 (7.8)	44.7 (9.6)
*P* value	<.001	<.001	<.001
Preoperative ASES (SD)	40.3 (17.8)	38.9 (16.9)	43.3 (19.5)
Postoperative ASES (SD)	81.4 (18.1)	81.0 (16.9)	82.4 (20.5)
*P* value	<.001	<.001	<.001
Preoperative SST (SD)	28.9 (22.0)	26.6 (21.2)	33.9 (23.3)
Postoperative SST (SD)	72.5 (24.0)	69.3 (23.7)	79.4 (23.6)
*P* value	<.001	<.001	<.001

*P-UE*, patient reported outcomes measurement information system; Upper Extremity; *ASES*, American Shoulder and Elbow Surgeons score; *SST*, Simple Shoulder Test; *SD*, standard deviation.

### Complications, readmission and revision rates

There were no significant differences between the two cohorts with respect to 90-day complications and revision rates (*P* = .34) (Table II). There were two complications within the Medicare cohort. One patient had a prosthetic joint infection treated with two-stage revision, and the second patient had prosthetic shoulder instability (secondary to rotator cuff tear) and was revised to rTSA. Both patients demonstrated complete resolution of their symptoms and complications following revision surgery.

## Discussion

The primary aim of this study was to determine if insurance provider, namely Medicare vs. private insurance displayed a difference in PROMs, complication, and revision rate after SA. We found no difference in PROMs for patients with Medicare and private insurance preoperatively or with regards to pre- and postoperative P-UE, ASES, and SST scores (*P* > .05). Furthermore, when determining if patients achieved the MCID after SA, insurance payor had no impact on patients achieving this clinical benchmark for each of the PROMs.^12^^,^^35^

It is generally believed that patients with Medicare insurance carriers are generally older and have higher rates of comorbidities when compared to patients covered by private insurance and require greater lengths of stay post total joint arthroplasty.^9^^,^^14^^,^^22^ In our study, the Medicare cohort was 9 years older than the private payor cohort. The comorbidity profile was significant for higher proportion of diabetes mellitus and overall higher CCI in the Medicare cohort compared to the private payor cohort, which has been reported previously. However, these aforementioned differences did not affect the short-term outcomes in our study. There have been mixed reports regarding the impact of insurance payor on outcomes after SA. Baettig et al evaluated 505 patients undergoing SA and rotator cuff repair surgery to further investigate what factors are associated with higher patient satisfaction outcomes.^3^ They found that patients with private insurance reported higher patient satisfaction than those covered by statutory insurance (not medicare, *P* = .0001).^3^ Lansdown et al evaluated PROMs for 438 patients covered by Medicare, Medicaid, and private insurance that underwent aTSA, rTSA, and hemiarthroplasty (HA).^17^ They found that Medicaid patients reported worse preoperative ASES scores relative to Medicare (8.7 vs. 13.1, *P* = .031), in addition to worse final ASES scores for Medicaid patients at 2-year follow-up relative to the private insurance cohort (66.1 vs. 78.3, *P* = .023).^17^ No differences were reported regarding ASES composite score difference from pre-to postoperative period for Medicaid, Medicare, and private insurance cohorts (*P* = .36). It is important to note that the Medicare and private insurance cohorts differed in the number of HA (*P* < .001) patients included, a procedure known to demonstrate lower PROMs when compared to TSA.^7^^,^^17^ Additionally, only 66% of patients enrolled in the study were included in the final results, considerably raising the potential for nonresponse bias.^17^ Sabesan et al reported the effect of insurance payor on outcomes of patients undergoing SA.^28^ They evaluated 120 patients with private insurance, 51 patients with Medicare, and 23 patients covered by the Veteran Affairs Care Program. Except for the higher Constant score in the private payor group (*P* = .029), they found no difference in PROMs used for evaluation, which included ASES (*P* = .118), SST (*P* = .218), UCLA shoulder scale (0.276), and shoulder pain and disability index (*P* = .676).^28^ Potential reasons for differences in results among these studies include differences in demographics among the patient cohorts. Our results have proven similar to previous studies; however, they are more robust by removing confounding variables, such as HA and resurfacing from the cohorts, directly comparing Medicare vs. private insurance. Additionally, we utilized P-UE, a survey specifically suited for determining the functional status of a patient’s UE that reduces survey burden for patients and also determined if any appreciable difference surpasses the MCID. Furthermore, by ensuring our retention rate was greater than 80% we aimed to reduce the potential for nonresponse bias, a limiting factor in previous studies. This study has effectively demonstrated the equivocal outcomes for patients undergoing SA who are covered by Medicare and private insurance. The two cohorts were similar with respect to the extent of glenoid wear, SDI, and preoperative outcome scores. Although there were differences in the type of arthroplasty prevalence (aTSA vs rTSA), age, and medical comorbidity, it did not affect the outcomes between the two cohorts.

Medicare provides health coverage for millions of Americans and provides a greater level of perceived healthcare security in patients above the age of 65 years. This study demonstrates that having Medicare insurance does not affect patient outcomes after SA. Additionally, this study expands upon previously reported literature regarding the effect of insurance payor on patient-reported outcomes for patients undergoing SA. However, there are several important limitations of our study. First, external validity of these results may be limited as the data was obtained from a single institution in a metropolitan city. Second, PROMs are subject to potential biases from the patient perspective depending on when the survey is completed.^1^^,^^2^^,^^8^^,^^12^^,^^19^^,^^27^ Third, we do not have satisfaction data in these patients. Although outcome scores correlate with patient satisfaction, this may not be true in every case. It is possible that patient satisfaction may be different between the cohorts despite no difference in outcome scores. Lastly, we did not compare other insurance types (Veteran’s Affair and Medicaid) in this study.

## Conclusion

Our study demonstrates that, at a tertiary level academic institution in a metropolitan city, payor type does not have significant impact on achieving MCID or pre-to-postoperative improvements in PROMs after SA.

## Disclaimers:

Funding: No outside funding or grants were received in support of the completion of this study.

Conflict of interest: The authors of this paper certify that they have NO affiliations with or involvement in any organization or entity with any financial or non-financial interests pertinent to the subject matter discussed in this manuscript.
